# Retraction Note: m^6^A demethylase ALKBH5 inhibits pancreatic cancer tumorigenesis by decreasing WIF-1 RNA methylation and mediating Wnt signaling

**DOI:** 10.1186/s12943-025-02432-5

**Published:** 2025-08-26

**Authors:** Bo Tang, Yihua Yang, Min Kang, Yunshan Wang, Yan Wang, Yin Bi, Songqing He, Fumio Shimamoto

**Affiliations:** 1 Department of Hepatobiliary Surgery, The First Affiliated Hospital of Guangxi Medical University, Nanning, Guangxi 530021 People’s Republic of China; 2 Center of Reproductive Medicine, The First Affiliated Hospital of Guangxi Medical University, Nanning, Guangxi 530021 People’s Republic of China; 3 Department of Radiation Oncology, the First Affiliated Hospital of Guangxi Medical University, Nanning, Guangxi 530021 People’s Republic of China; 4 Department of Clinical Laboratory, The Second Hospital of Shandong University, 247 Beiyuan Street, Jinan, Shandong 250033 China; 5 Department of Health Sciences, Hiroshima Shudo University, Hiroshima, 731-3195 Japan

**Retraction Note: Molecular Cancer 19, 3 (2020)**

**https://doi.org/10.1186/s12943-019-1128-6**

The Editor-in-Chief has retracted this article. After publication, concerns were raised regarding highly similar images between this article and earlier publications, specifically:

- Fig. 2J WT Gem left image appears to overlap with Fig. 3A Low-grade glioma middle and left images in [1];
- Fig. 4D Vector Migration image appears to overlap with Fig. 5D pSuper and shCUL7 #3 images in [2];
- Fig. 4D ALKBH5 Migration image appears to overlap with Fig. 8 Control TGF-β1- Migration and thalidomine TGF-β1- Invasion images in [3], as well as all four shCUL4A images in Fig. 7C of [4].

Additionally, some of the primer sequences used in this study appear to have different targets than those stated in Table S2.

The Editor-in-Chief therefore no longer has confidence in the presented data.

Yihua Yang agrees with this retraction. Bo Tang, Min Kang, Yunshan Wang, Yan Wang, Yin Bi and Songqing He have not responded to any correspondence from the editor or publisher about this retraction. The publisher has been unable to obtain a current email address for Fumio Shimamoto.
